# The role of cannabinoids in chronic pain management: clinical insights and challenges

**DOI:** 10.1016/j.bjane.2024.844523

**Published:** 2024-05-31

**Authors:** André P. Schmidt

**Affiliations:** a Hospital de Clínicas de Porto Alegre (HCPA), Serviço de Anestesia e Medicina Perioperatória, Porto Alegre, RS, Brazil; b Santa Casa de Porto Alegre, Serviço de Anestesia, Porto Alegre, RS, Brazil; c Hospital Nossa Senhora da Conceição (HNSC), Serviço de Anestesia, Porto Alegre, RS, Brazil; d Universidade Federal do Rio Grande do Sul (UFRGS), Programa de Pós-graduação em Ciências Pneumológicas, Porto Alegre, RS, Brazil; e Universidade Federal do Rio Grande do Sul (UFRGS), Programa de Pós-graduação em Ciências Cirúrgicas, Porto Alegre, RS, Brazil; f Faculdade de Medicina da Universidade de São Paulo (FMUSP), Programa de Pós-Graduação em Anestesiologia, Ciências Cirúrgicas e Medicina Perioperatória, São Paulo, SP, Brazil

Cannabinoid-based therapies have garnered significant attention as potential treatments for various types of pain, including chronic pain, neuropathic pain, and pain associated with cancer and neurological diseases. Phytocannabinoids, the active compounds found in cannabis plants, interact with the endocannabinoid system in the human body, playing a crucial role in modulating pain and inflammation.^1^ The most well-known phytocannabinoids are tetrahydrocannabinol (THC) and cannabidiol (CBD). The discovery of phytocannabinoids and their effects enabled the design of a myriad of synthetic cannabimimetic compounds that served as the basis for several groundbreaking studies in the last few years.^2^^,^^3^

Cannabinoids exert their analgesic effects primarily by activating cannabinoid receptors (CB1 and CB2) in the endocannabinoid system. CB1 receptors, predominantly located in the central nervous system, display critical roles in numerous functions and are associated with the well-known psychotropic effects of THC. Notably, CB1 receptors are located on neuronal circuits along the pain pathways, especially in the dorsal horn of the spinal cord.^4^ In contrast, CB2 receptors, mainly found in peripheral tissues and immune cells, are highly induced in microglia following injury and associated with anti-inflammatory and antinociceptive effects.^5^

The elucidation of the roles of the endocannabinoid system in pain modulation has been facilitated by animal models that employed a variety of sophisticated methodologies, including genetic knockout techniques and comprehensive pharmacological investigations.^2^^,^^6^^,^^7^ In a previous systematic review and meta-analysis of animal studies using inflammatory and neuropathic pain paradigms, CB1 and CB2 receptor agonists consistently decreased pain behaviors in inflammatory and nerve-injury models.^8^ Importantly, considering the complexity of multidimensional clinical pain, there is indeed a significant discordance between the apparent robust effect of cannabinoids in animal models compared with mixed evidence from human studies of cannabis.^9^

Several clinical trials and meta-analyses have investigated the efficacy of cannabinoids in pain management.^10^^,^^11^ Studies have shown that cannabinoids can provide moderate pain relief in chronic conditions, but these reports have provided mixed results on efficacy for chronic pain. A previous consensus from the National Academies of Sciences, Engineering, and Medicine has shown evidence of a significant reduction in chronic pain with the use of cannabis or cannabinoids.^12^ In a 2018 systematic review of clinical trials, there was moderate evidence that cannabis reduced chronic pain by 30%, but the rate of adverse events was relatively high; the number needed to treat was 24, and the number needed to harm was 6.^13^ A 2022 systematic review has investigated the effects of various doses and concentrations of synthetic and plant-extracted cannabinoids.^10^ This study found small and short-term improvements in neuropathic chronic pain, however conclusions from this review are limited by the heterogeneity of various cannabis products and the lack of adequate studies on specific preparations and patient populations. Of note, cannabis was used as an adjunct to other pain therapies in most studies.

While numerous studies have examined the utility of medical cannabis for managing chronic pain, most published data are of low to moderate quality due to small sample sizes, short follow-up periods, and non-blinded or non-randomized study designs. Additionally, most studies do not use a standardized dose or route of administration, and the chronic pain populations studied vary by the etiology of the pain. Given the complexity of the plant, lack of product quality control, and numerous routes of administration, conclusive statements on the actual analgesic benefits of cannabinoids still cannot be made.

While cannabinoids offer potential analgesic benefits, their use is associated with various side effects, including dizziness, dry mouth, fatigue, nausea, vomiting, drowsiness, and psychoactive effects, primarily due to THC.^14^ The risk of dependence and cognitive impairment also raises concerns, particularly with long-term use.^15^ CBD, a non-psychoactive cannabinoid, presents a more favorable safety profile and is being investigated for its anti-inflammatory and analgesic properties without the psychoactive effects.^13^^,^^14^

Importantly, the legal status of cannabinoid products varies globally, affecting their accessibility and clinical use. In some regions, cannabinoids are available only through special access schemes or clinical trials. Regulatory hurdles and variability in product quality and potency also pose significant challenges to their widespread adoption in pain management.

Ongoing research aims to better understand the mechanisms of cannabinoids and optimize their therapeutic use. This includes exploring the synergistic effects of different cannabinoids, developing formulations with improved safety profiles, and identifying patient populations that may benefit the most from cannabinoid therapy. Additionally, more high-quality randomized controlled trials are needed to establish standardized dosing guidelines and long-term safety data.

In this issue of the *Brazilian Journal of Anesthesiology*, a task force from the São Paulo State Society of Anesthesiology (SAESP) shares its general recommendations for using cannabinoid products for pain management.^16^ The authors conducted a narrative review using the Delphi method and requiring a minimum agreement of 60% among panelists. Overall, the document reinforced the potential of cannabinoids in some types of chronic pain management but underlined the importance of cautious prescription. Patients exhibiting poor therapeutic responses to established protocols or demonstrating intolerance to recommended management may be considered as potential candidates for cannabinoids, which should be prescribed by physicians experienced in handling these substances. Of note, the SAESP task force concluded that the use of cannabinoids for treating acute pain or cancer-related pain is still not supported by current scientific evidence.

By further analyzing the present literature review, we observe that there is a need for more systematic and rigorous research on cannabinoids for the management of pain to determine their safety and efficacy. Research on dosing procedures and product characteristics, as well as how these factors may impact other clinical outcomes, is crucial. More consistent evidence is needed to inform policy changes and patient/physician education, aiming to minimize potential risks and optimize benefits for patients seeking cannabinoids as an alternative treatment.

In summary, cannabinoid-based therapies hold promise for pain management, particularly in conditions where conventional treatments have displayed limited efficacy. However, balancing the benefits with potential risks and navigating regulatory landscapes remain key challenges. Continued research and well-designed clinical trials will be essential to fully integrate cannabinoids into mainstream pain management practices.

## Declaration of competing interest

The author declares no conflicts of interest.
